# Creating and facilitating change for Person‐Centred Coordinated Care (P3C): The development of the Organisational Change Tool (P3C‐OCT)

**DOI:** 10.1111/hex.12631

**Published:** 2017-11-15

**Authors:** Jane Horrell, Helen Lloyd, Thavapriya Sugavanam, James Close, Richard Byng

**Affiliations:** ^1^ Plymouth University Peninsula Schools of Medicine and Dentistry NIHR CLAHRC, South West Peninsula (PenCLAHRC) Plymouth UK; ^2^ Nuffield Department of Orthopaedics Rheumatology & Musculoskeletal Sciences (NDORMS) University of Oxford Oxford UK

**Keywords:** barriers, care, centred, coordinated, facilitators, general, implementation, person, practice

## Abstract

**Background:**

Person Centred Coordinated Care (P3C) is a UK priority for patients, carers, professionals, commissioners and policy makers. Services are developing a range of approaches to deliver this care with a lack of tools to guide implementation.

**Methodology:**

A scoping review and critical examination of current policy, key literature and NHS guidelines, together with stakeholder involvement led to the identification of domains, subdomains and component activities (processes and behaviours) required to deliver P3C. These were validated through codesign with stakeholders via a series of workshops and cognitive interviews.

**Results:**

Six core domains of P3C were identified as follows: (i) my goals, (ii) care planning, (iii) transitions, (iv) decision making (v), information and communication and (vi) organizational support activities. These were populated by 29 core subdomains (question items). A number of response codes (components) to each question provide examples of the processes and activities that can be actioned to achieve each core subdomain of P3C.

**Conclusion:**

The P3C‐OCT provides a coherent approach to monitoring progress and supporting practice development towards P3C. It can be used to generate a shared understanding of the core domains of P3C at a service delivery level, and support reorganization of care for those with complex needs. The tool can reliably detect change over time, as demonstrated in a sample of 40 UK general practices. It is currently being used in four UK evaluations of new models of care and being further developed as a training tool for the delivery of P3C.

## 1 INTRODUCTION

The current UK fiscal climate is demanding greater efficiency and cost‐saving across public sector organizations. The NHS in particular is facing unrivalled challenges to do more with less and deliver better quality and more efficient care whilst reducing deficits.^1^, ^2^ It is in this context that a move away from disease‐based models towards a more effective, integrated, and person‐centred approach is perceived as a way to reorganize service delivery. This is particularly relevant for people with long‐term conditions (LTCs), multiple LTCs and people with multimorbidity; the number of which is forecast to rise from 1.9 million in 2008 to 2.9 million in 2018.^3^

Person Centred Care (PCC) is an approach to patients that embodies an individual's right to self‐determination and highlights their role as an equal partner in the care exchange.^4^ Recent work has identified care coordination rather than organizational integration as one of the essential components for the delivery of PCC.^5^, ^6^, ^7^, ^8^ This means that changes in the ways professionals work are required,^9^ as it cannot be effectively delivered in a system that is confused, fragmented and lacking in continuity.^7^, ^10^ In a European context, Ekman et al have provided a guide on how to approach PCC through the development of three routines based around (i) eliciting the individuals narrative, (ii) the cocreation of a plan of care and (iii) documenting this plan within a care plan.^4^ Lloyd has expanded this into four routines to fully encompass the needs of those with complex needs; (iv) an agreement to act in conjunction with the person and other professionals to coordinate care.^11^

These two key concepts brought together reflect a possible way in which to achieve better outcomes of care for individuals: Person Centred and Coordinated Care (P3C). We define P3C as:

Care and support that is guided by and organized effectively around the needs and preferences of individuals.

11

In detail, the following table provides a breakdown of the three elements of the current definition of Person Centred Coordinated Care (Table 1):

**Table 1:** Three elements of the current definition of P3C

Person Centred Care	The cocreation of care between the patients, their family and informal carers, and health professionals. This definition is becoming widely used by many international organizations including the WHO, and has been translated into a proven approach and used at the Gothenburg University Centre for Person Centred Care (GPCC). Person‐centred care strives to see an individual as bio‐psycho‐social whole, as a person and not an illness or a collection of conditions
Resources	Psycho‐social and environmental resources are non‐clinical and have a community focus. This is commonly being referred to as “Community‐centred approaches” that complement other types of interventions that focus more on individual care and behaviour change or on developing sustainable environments. These approaches acknowledge the importance of social capital for health and well‐being to flourish
Coordinated Care	Care coordination is the deliberate organisation of patient care activities between two or more participants (including the patient) involved in a patient's care to facilitate the appropriate delivery of healthcare services. Organizing care involves the marshalling of personnel and other resources needed to carry out all required patient care activities and is often managed by the exchange of information among participants responsible for different aspects of care. From a person or family perspective, care coordination is any activity that helps ensure that the individual's needs and preferences for health services and information sharing across people, functions and sites are met over time

P3C highlights the patient as an “expert,” with access to both individual and environmental resources, and around all of which care should be coordinated. Anchored in the National Voices “I” Statements,^12^ P3C places an emphasis on the individual and reflects what is important to them in relation to their care and support needs. This approach holds the promise of improved outcomes and experiences through the setting and attainment of personal goals based on the values and preferences of the individual (elicited through shared decision making).^13^, ^14^ The logic therefore follows that this approach produces care and support that is tailored to the individual and is more efficient at reducing waste and duplication.

Implementation of new models that seek to provide more integrated care has been hampered by conceptual confusion and a lack of practical guidance. As a result, this care is rarely delivered or implemented in a consistent manner.^4^ The UK House of Care model^15^ was developed with the aim of designing a partnership delivery model for Person Centred Care (PCC), encompassing coordinated services and available to all people with long‐term conditions. It was established to move away from a single disease‐focused reactive system towards a more pre‐emptive, holistic view of the person that assigned an active role for patients. Its goal is to drive a whole system approach, based on the understanding that critical elements are required to deliver care in this way. Whilst the model provides a summary of areas where changes are required, few sites within the UK have achieved the implementation of the complete paradigm.^15^ This may be in part due to the abstract constructs that abound the policy literature of this area, and which are difficult to implement without the specific detail of processes of change. For example, Coulter et al^16^ state that the most robust barrier to the delivery of PCC is cultural change. This is a very real obstacle, but “culture change” within this model is not articulated in a way that identifies the behaviours and processes necessary to change cultures of practice.

A shift towards P3C also brings with it a requirement to measure and guide the development of services whilst considering organizational context (eg rural/suburban/urban) and how this influences the design and configuration of services.^6^, ^17^ However, at present there are no comprehensive tools that can achieve this within health and social care settings.^6^, ^17^ Our scoping exercise to identify ways in which P3C can be achieved failed to identify guidance that was sufficiently detailed to support implementation. We found evidence of only one co‐created quality improvement organizational tool that encompassed an element of PCC.^18^ This tool is intended for use in Australian general practice, and given the UK push for integrated health and social care, there remains the need for a tool which can be implemented across a range of services.

### 1.1 Aim

Our aim was to develop a practical tool to support organizations and practitioners to provide personalized and coordinated care for people with multimorbidity. This tool is based in on the principles of promoting person‐centred relationships with service users and between practitioners, and highlights how organisations can support its achievement.^19^, ^20^

## 2 METHOD

**Figure 1:**
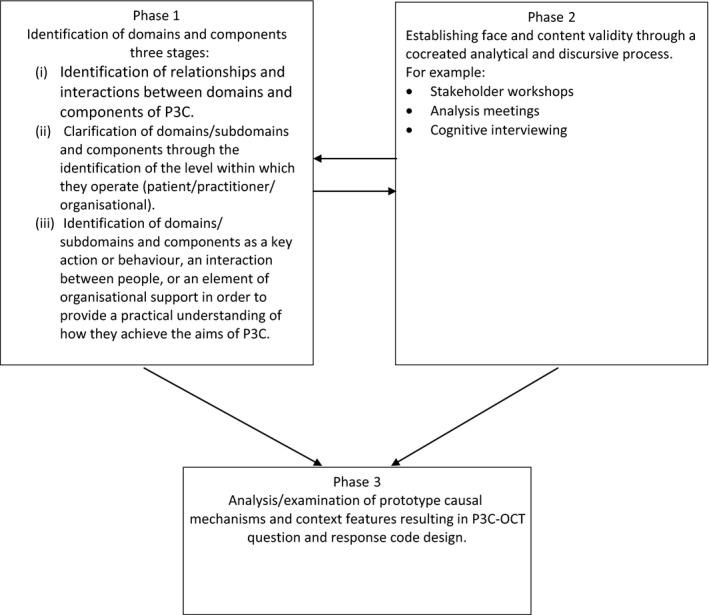
Three‐phase methodology

The development of the Person Centred Coordinated Care Organisational Change Tool (P3C‐OCT) transpired in response to the requests of our local stakeholders (commissioners, practitioners, members of the Peninsula Public Involvement Group [PenPIG] and the South West Academic Health Services Network [SWAHSN]). The tool evolved from work to develop a taxonomy which aimed to identify, clarify and define the domains, subdomains and components hypothesized as necessary for P3C.^21^ Its advancement was iterative and progressed during three phases (see Figure 1).

### 2.1 Phase One: Identification and allocation of domains and components into actions, roles and responsibilities across the organizational structure

Phase one consisted of three stages. Stage (i) comprised of the identification of relationships and interactions between domains, subdomains and component activities of P3C (eg Personalised Care Planning is a core domain but also potentially acts as a component of the My Goals domain).

Stage (ii) Subdomains/components were assigned to an operating interface (ie where and with whom they were most likely to take place). This was achieved through splitting them into actions and associated roles and responsibilities (patient/practitioner/organisational). Stage (iii) culminated in the specification of where the component activities would predominantly function: (1) clinical/ patient interface, (2) functional integration (information systems/ IT tools) and (3) organisational systems. This enabled the progression of a practical understanding of how components potentially interact to support the achievement of the subdomains of P3C.

### 2.2 Phase Two: Validation and endorsement of components, and testing for relevance and readability

Phase two used codesign and ran concurrently with phases (1) and (3) to validate and endorse the clustering of domains, subdomains and components, and pilot test for readability and content validity. Literature and policy were repeatedly examined to ensure latest findings continued to be incorporated, and questions were interrogated and adapted (if necessary) to ensure domains and subdomains encompassed micro‐ and meso‐levels within the organisation. Further analytical work and cognitive interviewing^22^ with healthcare professionals explored the relevance of items and clarity of language and meaning. Feedback was used to improve question design and inform revisions, and map domains/subdomains and components to clinical practice and patient experience. Pilot data collected from General Practices (n = 40) were used to test the acceptability and meaningfulness of the tool on a wider sample. Free text boxes provided the opportunity to identify barriers and facilitators to professional interaction with the tool and allow for the suggestion and refinement of the tool.

Following pilot testing qualitative interviews and observations were used to further validate the tool within GP practices (n = 4). These practices were sampled on the basis of their summary OCT score to identify high/low scoring practices for care planning and care coordination. Selection was also guided by contextual features (practice size, rural/urban). Observations of patient/practitioner consultations (n = 6) were mapped against the emerging P3C‐OCT domains and components. Semi‐structured interviews were conducted with practitioners (n = 8) using the subdomains/domains as a framework for capturing the relevant elements of practitioner's understanding of delivering P3C.

A range of stakeholders were engaged using a workshop format from across Clinical Commissioning Groups (CCGs), clinicians, academics, voluntary organizations and patient representative groups to cross‐examine meaning, structure and language.

A further validation process involved mapping the identified P3C components and subdomains to the National Voices “I” Statements^12^; which were produced by a UK service user organization to describe what patients want from their care. Additional mapping was also undertaken against principles of P3C developed by the local patient involvement group PenPIG. This work enabled us to verify that the domains/ subdomains and components of P3C were relevant to patients and anchored within the aim of promoting a person‐centred and coordinated experience.

### 2.3 Phase Three: Design of questions and response codes

Phase three ran concurrently with phase 2 and comprised the development of questions and response codes iteratively in response to stakeholder guidance. Configurations of possible components (actions/behaviours etc.) were explored to ascertain how these acted as potential mechanisms to achieve each subdomain. This process continued until saturation was complete (ie no new configurations could be identified).

### 2.4 Ethics

Stakeholder involvement and the gathering of pilot data were approved by Plymouth University Faculty Research Ethics Committee.

### 2.5 Recruitment and procedure

#### 2.5.1 Stakeholder engagement for tool development

##### Patient representatives

Co‐design was achieved through two stakeholder workshops in conjunction with the PenCLAHRC Public Involvement Group (PenPIG). Potential participants were sent an invitatory email and study information sheet to help inform their decision to take part. Consent forms were completed and their right to withdraw explained. All participants were diagnosed with multiple LTCs.

##### Workshop with Health and Social Care professionals and patients

Feedback on question items was received as part of a wider workshop on outcome measures for P3C. Participants were known to the research group and were invited to take part either by email, telephone or in person.

##### Cognitive interviewing

Participants were recruited either through the academic team (n = 1) or from evaluation work where the tool was being piloted (n = 2). Participants were sent an invitation email and study information sheet to help inform their decision to take part. Consent forms were completed and their right to withdraw explained.

## 3 RESULTS

### 3.1 Development of the P3C‐OCT

The P3C‐OCT tool consists of 29 core questions across four operational levels: (i) person/practitioner interaction (person‐centred behaviours and activities), (ii) practitioner/practitioner interaction (coordination), (iii) organisational systems and support and (iv) information systems/IT support (see Appendix S1). There are a further 2 “text box” only questions for managers and practitioners to reflect on the use of the tool and assist in its further development, and a set of questions to tap organisational demographics. Core questions are allocated to operational level as follows:

1. Person‐Practitioner Interactions (11 questions): measuring aspects such as communication with patients to help them set and plan their goals.
2. Practitioner‐Practitioner Interactions (four questions): measuring aspects such as internal coordination of patient‐centred care and relationships with other organisations.
3. Organizational Systems & Support (12 questions): aspects such as staff training and measurement of patient experience.
4. Information systems/IT tools (four questions): aspects such as IT systems and telemedicine.

Within the above levels, there are 435 possible components/response codes which provide examples of how domains/subdomains (phrased as questions) can be achieved (see Table 2).

**Table 2:** Dimensions, subdimensions and the formation of P3C components into question items

Domain	No of question items in each domain	Subdomains	No of question items tapping each subdomain
My goals	6	Goal setting	2
		Empowerment and activation	3
		Self‐management	3
		Carer support	1
Decision making	2	Involvement in decision making	2
Care planning	14	The care plan	4
		Case management	7
		Single point of contact	3
		Care coordination	7
		Supporting people to stay at home	1
Information and communication	6	Relational continuity	2
		Information gathering/sharing	5
		Knowledge of patient/familiarity	1
Transitions	6	Continuity of care	6
Organisational processes and activities	8	Valuing physical and mental health equally	1
		Experience of care	1
		Longer appointment times	1
		Staff training	1
		Processes to address polypharmacy	1
		P3C leadership/culture change	4

### 3.2 Scoring the P3C‐OCT

The P3C‐OCT is scored with each of the 29 core questions which are equally weighted. Each of these 29 questions has both an objective component (eg component activities of P3C that are being delivered) and a subjective component (eg how well the respondent thinks these are working). Each question receives a maximum score of 20 points, with a maximum 10 points being allocated for the objective component, and likewise a maximum 10 points being allocated for the subjective component.

For scoring the objective component, the maximum allowable score (10) is divided by the possible number of activities (response codes). For example, with question 1, there are eight possible responses each of which is equally weighted. Thus, each activity receives a score of 1.25, and full activity (eg every activity is being performed) will score the maximum of 10. The scoring mechanism makes the assumption that evidence of activity is positive. Furthermore, any response in the “other” box is scored, as this is intended to be used to indicate other activities not covered by the standard responses.

Similarly, for the subjective component of each of the 29 questions, the maximum allowable score (10) is divided by the number of response codes. However, unlike the objective component (where responses are binary), the responses for the subjective component are on a 5‐point Likert scale (working very well; working well; requires some improvement; requires significant improvement; and not working/not relevant), and the scoring is therefore progressive. The minimum score is −10 (eg all activities are “not working”), and the maximum score is +10 (eg all activities are “working very well). The responses “not relevant,” “none” and “not working” are treated as equivalent.

According to this scoring mechanism, if all activities are being performed (+10 points for objective) but are “not working,” the subjective score will be −10. This results in an aggregate objective + subjective score of 0, so that evidence of an activity (and it not working) has the same score as not implementing an activity at all. See question example in Table 3 below:

**Table 3:** P3C‐OCT example question

Q4. In general, which of the following elements are included in the cocreated plan of care (this can either be in the form of a written document or a plan of working)?			
A lead coordinator	□	A List of medications and instructions for when to take	□
A contingency plan for crisis episodes or exacerbations of their condition	□	A date for review	□
A named person to contact in a crisis	□	Treatment Escalation Plan	□
An action plan to attain their health goals	□	Other (please specify)	□
An action plan to attain their social goals	□	*None*	□
Details of who is responsible for what	□	*Not relevant*	□
How well are your care plans working?		Comments (eg which aspects are working particularly well/not well):	
Working very well	□		
Working well	□		
Requires some improvement	□		
Requires significant improvement	□		
Not working	□		

Once scores have been derived for each question, they can be aggregated to derive a total score for the P3C‐OCT. The total score is normalized to 20 so that the overall score is out of a maximum of 20. Furthermore, scores can be derived for only objective components (eg a summary of activity towards P3C) or subjective components (eg how well things are working). Scores can be also derived according to domains/subdomains of P3C, by aggregating questions that correspond to these domains.

All questions follow the above schema, with a number of exceptions where the question format requires an idiosyncratic scoring. This is achieved in the most parsimonious manner. For instance, question 6 has two objective components (the second part is about using personal budgets), and these two components are aggregated (as if they were a single question) so that the maximum objective score remains equal to 10. Nonetheless, the “equally weighted” scoring mechanism has been retained so that all questions still retain a maximum of 10 points for objective and 10 points for subjective.

Feedback is delivered in the form of an interactive “dashboard” of results and a set of instructions to assist practices in its navigation. The dashboard provides full and complete set of responses to the P3C‐OCT. This automated scoring mechanism is provided at a number of different levels: (i) an aggregate result for total performance on the P3C‐OCT (out of 20) which may be presented in comparison with other practices if relevant or an internal benchmark for between time comparisons, (ii) an automated score for each of the individual 29 questions on the P3C‐OCT and (iii) an automated score for different domains of P3C. These scores can be presented in relation to aggregate scores of other organisations, allowing a comparison of scores to others.

There are a number of caveats that should be recognized when the data are presented in this manner. First, this aggregation overlooks much of the nuances and detail in the data. Second, for those areas where performance is below average, this may be influenced by factors that are beyond the organisation's control, such as interoperability and governance. Furthermore, the size and structure of the organisation may be a mediating factor on the scores, whereby (for instance) P3C may be implemented differently in large vs small organisations. Moreover, these scores can also be influenced by the subjective opinions of the person completing the P3C‐OCT.

The tool is designed to be completed in the form of a paper/electronic/or online document. In its current form, it is best suited to completion by organisational managers and clinical/service leads. Although one or two people may take responsibility for its completion, they will need to gather information from several key professionals (eg GP's, nurses, community matrons) and members of other teams which come together to provide multidisciplinary care. For this reason, questions may be circulated across the relevant professionals, or the tool can be completed as a team.

### 3.3 Validation

In response to stakeholder workshops and cognitive interviewing, a number of changes were made to the P3C‐OCT (see Table 4 for examples). In particular, changes related to ambiguous wording (for example, “care providers” was changed to paid care providers for clarity) and clarification on specific terminology. For example, originally, the tool referred only to a care plan. Feedback suggested that this was too restrictive and ignored the construct of care planning in terms of discussions with patients and colleagues.

**Table 4:** Example stakeholder feedback to improve question design and inform revisions

	Queries	Changes implemented
Clarity of Language	Need to define what we mean by “plan of care”/ “care plan document.” Care plan document does not allow for summaries written in notes‐ too formal and won't capture more informal/ subtle aspects of recording care planning/ goals discussions	Definitions added
	“People suitable for P3C”‐ what does this mean?	Replaced with “people who could benefit”
	“Which of the following elements are included in the plan of care.” Too rooted in idea of standardisation. In order to be person centred, elements included may vary across individual need. Rephrase so that it states “in general” which elements are included	Rephrased so that question states “in general, which elements are included”
	Care providers needs clarification‐ paid? Family/friends?	Rephrased to read “paid care providers”
	Clarification of what we mean by “implementation of specialist support services” what does this mean?	Added, for example intermediate/complex care teams
	Need to define telehealth, telecare, telemedicine to assure same understanding	Definitions added
	Question regarding people only having to tell their story once. This is unavoidable as changes happen	Rephrased to read “What do you do to ensure that a person doesn't have to tell their story repeatedly and unnecessarily?”
Missing response codes	Additional roles need to be added for who may take the lead for planning & coordination	Response codes added: Managerial lead Clinical lead Team coordinator
	Additional organisation needs to be added to partnership working	Response code added: Residential care homes
Streamlining of the tool	Completion too long and unwieldy	“Objective and subjective components within question design merged.”

Due to the size and complexity of the P3C‐OCT, many standard methods of psychometric validation are not applicable. First, it is not designed to be internally consistent (due to the multidimensional design) such as a standard questionnaire tool, precluding the use of Cronbach's alpha. Second, approaches such as principal component analysis are inappropriate due to the large number of items, requiring large sample sizes (which is challenging to achieve in a tool aimed at organisations). Furthermore, as an evaluation and implementation tool, flexibility is required in the model as it is likely that the configuration of components integral to P3C delivery will vary across settings in all but a core few (shared decision making, person‐centred goals and outcomes).

Instead, the primary validity of the P3C‐OCT is established by sensitivity to change. During our ongoing evaluation work, we have a cohort of 40 general practices that have completed the P3C‐OCT at two time points, approximately 6 months apart. These practices have been actively engaged with a number of schemes aimed at improving P3C, and aggregate scoring of these practices revealed a mean time 1 score of 5.8 (out of 20), and a time 2 score of 6.7. This mean difference of 0.9 improvement is significant at *P* = .034 on a paired t test (preliminary data; full publication of evaluation studies is undergoing preparation).

### 3.4 Pilot testing

The tool is currently being trialled across a number of South West evaluations. It has also been used to design, analyse and interpret organisational processes towards achieving P3C, and was used as a framework for the construction of questions to elicit the impact of change on practices opting out of QOF.^23^ Observations of patient/clinician consultations were successfully mapped to the emerging domains and components and further validated the tools content. Interview analysis showed that the components from the P3C‐OCT provided an effective framework for capturing the relevant elements of practitioners understanding of delivering P3C. To date, the tool has been piloted across 40 practices within Somerset as part of the Somerset Practice Quality Scheme (SPQS), and feedback workshops provided further data about how professionals interact with the tool.

## 4 DISCUSSION

The delivery of person‐centred coordinated care has been enmeshed in an environment of conceptual confusion and ambiguous language, resulting in a lack of tangible guidance on its implementation at an organizational level, and difficulties in real‐world application.^24^, ^25^ Components and domains of P3C identified in the literature range from broad themes to specific actions across domains which become unwieldy when combined into an assessment framework.^26^ Although aims of programmes appear similar, that is the reduction in fragmentation and the enhancement of continuity and coordination through the placing of the person at the centre of health‐care delivery,^25^ the processes through which to achieve these are less so.^27^The P3C‐OCT reflects the importance of committing resources to the development of policies and processes and adds to the consensus that multiple components are involved in its successful implementation.^28^ Crucially to the delivery of P3C, the tool supports organisations to better understand their own practice^29^ and to identify whether “I” statement and House of Care principles are being delivered.^12^

The P3C‐OCT unpicks the conceptual confusion of how to do P3C at an organisational level and provides guidance in a single toolkit. It is the first comprehensive evidence‐based tool that brings together a set of actions and behaviours to achieve the domains/ subdomains of P3C, and which can be implemented as a means to achieve routines evidenced as necessary to its accomplishment.^4^, ^20^ Whilst we recognize that we do not yet have a definitive picture of what “good” P3C looks like, we do know what core domains, subdomains and component activities can be implemented through the P3C‐OCT to provide the building blocks for organisations to learn and develop together.

The tool and its associated dashboard of results are intended to be interactive and to provide a space for practices to identify and debate P3C understandings and areas for development. It can also be used for reporting through internal and external benchmarking to track to the shifts in organisational and practitioner approaches over time and offer pathways towards further development. The P3C‐OCT provides a wealth of information across both individual and aggregated data sets and functions in four ways: (i) Individual data can be fed back to organisations, clinicians and healthcare professionals to help them understand what they are doing to deliver P3C, and what they can do to improve it. Not only does it reveal what practices and organisations are doing to implement P3C, but it also creates a dialogue between organisations/professionals to share and learn from each other. (ii) Individual data can also be fed forward to patients to help them understand the changes that organisations are implementing for P3C. (iii) Data from the tool can also be used by researchers to characterise P3C implementation and create an understanding of the facilitators/barriers to organisational implementation. (iv) Data can be used by health‐care managers and commissioners to help them understand how they can facilitate the commissioning and delivery of P3C. Often, barriers to implementing P3C are systemic and beyond the control of front‐line services. This tool is essential to gather this evidence and help to identify problems, and enhance the dialogue between services, service providers, commissioners and researchers.

The P3C‐OCT is part of a suite of tools developed by our collaboration of academics and key stakeholders in the South West, consisting of our team at Plymouth University (Primary Care), the Peninsula CLAHRC and the Academic Health Science Network. This has also involved the development of a patient experience measure^30^ to embed the patient voice at the heart of service redesign.

### 4.1 Implications for future research and practice

#### 4.1.1 Further validation of the domains and components of P3C

Currently, we have little evidence for the optimal configurations of behaviours, interactions and system support to produce P3C and further research using the OCT tool or similar approaches is required to address this. Further work also needs to consider fully the impact of contextual features (eg practice size, rural/urban) and their impact on the achievement and implementation of components.

#### 4.1.2 Understanding and development of how the tool is used in practice

The development of the P3C OCT will also benefit from widespread in‐practice use. Given the wealth of data collected for each completion, the dashboard requires refinement to maximize its ability to be user‐friendly. Work is currently being undertaken to deliver the dashboard on a web‐based platform. This will allow users to log in and access their results and will enhance its interactivity. Feedback from professionals has also highlighted a wish for a portfolio of generalizable intelligence to guide improvements to practice; it is envisaged that this too could form part of the web‐based platform.

To advance the development of the tool, consideration also needs to be given to its ability to adapt to the ever‐changing landscape of health care. For example, pilot data suggest that practices are increasingly employing new types of health‐care professionals such as Health & Wellbeing coordinators. In order for the tool to remain relevant to practice, collaborative work with stakeholders needs to continue to ensure changes are incorporated in a timely manner.

## 5 CONCLUSIONS

Implementing P3C is a complex and multifaceted intervention that requires support and action at all levels. The P3C‐OCT operates at a practice‐based organisational level to assist organisations and practitioners to critically reflect on practice and service development. It encompasses organisational domains and components of P3C and provides the tools to build further on Ekman^4^ and Lloyd's^20^ routines centring around active listening, shared decision making and coordinated working around a documented cocreated plan of care. The tool aims to guide organisations through a range of concrete actions, interactions and system enablers towards P3C.

The tool is currently being tested and used as a monitoring and change instrument in four evaluations of P3C across a range of UK sites and models of care. Pilot testing will continue and feedback will be used to adapt and improve the tool. We theorise that ongoing interrogation of the interaction between domains/subdomains (question items) and components (response codes) from implementation data will allow the development of a more comprehensive theory of what works for whom and in what situations to best accomplish P3C.

## AUTHORS’ CONTRIBUTIONS

Ms Jane Horrell and Dr Helen Lloyd contributed to the design, analysis and interpretation of data. Dr James Close contributed to analysis and the design of the feedback dashboard. Dr Thavapriya Sugavanam contributed through her involvement in the wider programme of P3C work. Professor Richard Byng was responsible for developing the initial concept and supported the development of the tool. All five authors contributed to the writing of the paper.
