# Supplementary material for: Creating and facilitating change for Person‐Centred Coordinated Care (P3C): The development of the Organisational Change Tool (P3C‐OCT)
Source: Health Expect. 2017 Nov 15;21(2):448–56. doi: 10.1111/hex.12631 (PMC5867330; doi:10.1111/hex.12631)
Supplement: Supplementary file 1 [file HEX-21-448-s001.docx]

Appendix 1

Question mapping to Domain/ Subdomain

| **Question Number** | **Level** | **Domain** | **Subdomain** | **Primary Domain/s question taps** | **Secondary Domain/s question taps** |
| --- | --- | --- | --- | --- | --- |
| 1 | **Person-Practitioner** | Transitions | Continuity of Care | X |  |
|  |  | Information and Communication | Information gathering/sharing | X |  |
|  |  | Information and Communication | Knowledge of patient/familiarity | X |  |
|  |  | Care Planning | Care coordination |  | X |
|  |  | Care Planning | Single point of contact |  | X |
|  |  | Care Planning | Care plan |  | X |
|  |  | Organisational process activities | Experience of care |  | X |
| 2 | **Person-Practitioner** | My Goals Outcomes | Empowerment/activation | X |  |
|  |  | My Goals Outcomes | Self-management | X |  |
|  |  | Information and Communication | Information gathering/sharing | X |  |
|  |  | My Goals Outcomes | Goal Setting |  | X |
|  |  | My Goals Outcomes | Carer support |  | X |
|  |  | Care Planning | Care plan |  | X |
| 3 | **Person-Practitioner** | Care Planning | Care plan | X |  |
|  |  | Care Planning | Care coordination | X |  |
|  |  | My Goals Outcomes | Empowerment/activation |  | X |
|  |  | Information and Communication | Information gathering/sharing |  | X |
|  |  | Information and Communication | Knowledge of patient/familiarity |  | X |
|  |  | Transitions | Continuity of Care |  | X |
| 4 | **Person-Practitioner** | Care Planning | Care plan | X |  |
|  |  | My Goals Outcomes | Goal Setting |  | X |
|  |  | My Goals Outcomes | Self-management |  | X |
|  |  | Care Planning | Care coordination |  | X |
|  |  | Care Planning | Supporting people to stay at home |  | X |
|  |  | Information and Communication | Information gathering/sharing |  | X |
|  |  | Information and Communication | Relational continuity |  | X |
| 5 |  | Care Planning | Care coordination | X |  |
|  |  | Information and Communication | Relational continuity |  | X |
|  |  | Care Planning | Single point of contact |  | X |
|  |  | Care Planning | Case management |  | X |
|  |  | Transitions | Continuity of Care |  | X |
| 6 |  | Decision Making | Involvement in decision making | X |  |
|  |  | My Goals Outcomes | Goal Setting |  | X |
|  |  | My Goals Outcomes | Empowerment/activation |  | X |
|  |  | My Goals Outcomes | Self-management |  | X |
|  |  | Information and Communication | Information gathering/sharing |  | X |
|  |  | Organisational process activities | Longer appointment times |  | X |
|  |  | Organisational process activities | Experience of care |  | X |
| 7 |  | My Goals Outcomes | Goal Setting | X |  |
|  |  | My Goals Outcomes | Empowerment/activation |  | X |
|  |  | Care Planning | Care plan |  | X |
|  |  | Information and Communication | Information gathering/sharing |  | X |
|  |  | Organisational process activities | Staff training |  | X |
| 8 |  | My Goals Outcomes | Goal Setting | X |  |
|  |  | Care Planning | Care plan |  | X |
|  |  | Information and Communication | Information gathering/sharing |  | X |
|  |  | Organisational process activities | P3C leadership/ supporting culture change |  | X |
| 9a |  | My Goals Outcomes | Empowerment/activation | X |  |
|  |  | My Goals Outcomes | Self-management | X |  |
|  |  | Care Planning | Care plan |  | X |
|  |  | Information and Communication | Information gathering/sharing |  | X |
|  |  | Organisational process activities | Experience of care |  | X |
| 9b |  | My Goals Outcomes | Empowerment/activation | X |  |
|  |  | My Goals Outcomes | Self-management | X |  |
|  |  | Care Planning | Care plan |  | X |
|  |  | Information and Communication | Information gathering/sharing |  | X |
| 10 |  | Organisational process activities | Valuing Physical & mental health equally | X |  |
|  |  | Care Planning | Care plan |  | X |
|  |  | Care Planning | Care coordination |  | X |
|  |  | Organisational process activities | Longer appointment times |  | X |
| 11 |  | My Goals Outcomes | Carer support | X |  |
|  |  | My Goals Outcomes | Goal Setting |  | X |
|  |  | My Goals Outcomes | Empowerment/activation |  | X |
|  |  | My Goals Outcomes | Self-management |  | X |
|  |  | Decision Making | Involvement in decision making |  | X |
|  |  | Information and Communication | Information gathering/sharing |  | X |
|  |  | Organisational process activities | Experience of care |  | X |
|  |  | Organisational process activities | Valuing Physical & mental health equally |  | X |
|  |  | Organisational process activities | Supporting people to stay at home |  | X |
| 12 |  | Care Planning | Care coordination | X |  |
|  |  | Care Planning | Case management | X |  |
|  |  | Transitions | Continuity of care | X |  |
|  |  | Information and Communication | Information gathering/sharing | X |  |
|  |  | Information and Communication | Relational continuity |  | X |
|  |  | Information and Communication | Knowledge of patient/familiarity |  | X |
|  |  | Organisational process activities | P3C leadership/ supporting culture change |  | X |
| 13 |  | Care Planning | Care coordination | X |  |
|  |  | Care Planning | Case management | X |  |
|  |  | Transitions | Continuity of care | X |  |
|  |  | Information and Communication | Information gathering/sharing | X |  |
|  |  | Information and Communication | Relational continuity |  | X |
|  |  | Information and Communication | Knowledge of patient/familiarity |  | X |
|  |  | Organisational process activities | P3C leadership/ supporting culture change |  | X |
| 14 |  | Care Planning | Care coordination | X |  |
|  |  | Care Planning | Case management | X |  |
|  |  | Transitions | Continuity of care | X |  |
|  |  | Information and Communication | Information gathering/sharing | X |  |
|  |  | Information and Communication | Relational continuity | X |  |
|  |  | Care Planning | Care plan |  | X |
|  |  | Information and Communication | Knowledge of patient/familiarity |  | X |
|  |  | Organisational process activities | P3C leadership/ supporting culture change |  | X |
| 15 |  | Care Planning | Care plan | X |  |
|  |  | Care Planning | Care coordination | X |  |
|  |  | Transitions | Continuity of Care | X |  |
|  |  | Information and Communication | Information gathering/sharing |  | X |
|  |  | Information and Communication | Knowledge of patient/familiarity |  | X |
| 16 |  | Care Planning | Case management | X |  |
|  |  | Care Planning | Care coordination |  | X |
|  |  | Information and Communication | Relational continuity |  | X |
| 17 |  | Organisational process activities | Experience of care | X |  |
|  |  | My Goals Outcomes | Carer support |  | X |
| 18 |  | Care Planning | Case management | X |  |
|  |  | Care Planning | Care coordination |  | X |
|  |  | Information and Communication | Information gathering/sharing |  | X |
|  |  | Information and Communication | Knowledge of patient/familiarity |  | X |
|  |  | Transitions | Continuity of Care |  | X |
| 19 |  | Care Planning | Case management | X |  |
|  |  | Care Planning | Single point of contact |  | X |
|  |  | Care Planning | Care coordination |  | X |
|  |  | Organisational process activities | Supporting people to stay at home |  | X |
|  |  | Information and Communication | Information gathering/sharing |  | X |
| 20 |  | Organisational process activities | Longer appointment times | X |  |
|  |  | Organisational process activities | P3C leadership/ supporting culture change |  | X |
| 21 |  | Decision Making | Involvement in decision making | X |  |
|  |  | My Goals Outcomes | Empowerment/activation |  | X |
|  |  | My Goals Outcomes | Self-management |  | X |
|  |  | Organisational process activities | Experience of care |  | X |
| 22 |  | Care Planning | Case management | X |  |
|  |  | Care Planning | Care coordination |  | X |
|  |  | Information and Communication | Information gathering/sharing |  | X |
| 23 |  | Organisational process activities | Staff training | X |  |
|  |  | Organisational process activities | P3C leadership/ supporting culture change | X |  |
| 24 |  | Organisational process activities | P3C leadership/ supporting culture change | X |  |
| 25 |  | Organisational process activities | Supporting people to stay at home | X |  |
|  |  | Transitions | Continuity of Care | X |  |
|  |  | Care Planning | Care plan |  | X |
|  |  | Care Planning | Self-management |  | X |
|  |  | Care Planning | Empowerment/activation |  | X |
|  |  | Care Planning | Care coordination |  | X |
|  |  | Organisational process activities | P3C leadership/ supporting culture change |  | X |
| 26 |  | Organisational process activities | Processes to address polypharmacy | X |  |
|  |  | My Goals Outcomes | Self-management |  | X |
|  |  | Care Planning | Case management |  | X |
|  |  | Information and Communication | Information gathering/sharing |  | X |
| 27 |  | Organisational process activities | P3C leadership/ supporting culture change | X |  |
|  |  | Organisational process activities | Experience of care |  | X |
| 28 |  | My Goals Outcomes | Self-management | X |  |
|  |  | My Goals Outcomes | Empowerment/activation | X |  |
|  |  | Organisational process activities | P3C leadership/ supporting culture change |  | X |
| 29 |  | Care Planning | Care plan | X |  |
|  |  | Care Planning | Care coordination | X |  |
|  |  | Organisational process activities | P3C leadership/ supporting culture change | X |  |
|  |  | Care Planning | Empowerment/activation |  | X |
|  |  | Care Planning | Case management |  | X |
|  |  | Information and Communication | Information gathering/sharing |  | X |
|  |  | Information and Communication | Knowledge of patient/familiarity |  | X |
| 30 |  | None assigned N/A | None assigned N/A |  |  |
| 31 |  | None assigned N/A | None assigned N/A |  |  |
